# Inhibitory and Bactericidal Potential of Crude Acetone Extracts of *Combretum molle* (Combretaceae) on Drug-resistant Strains of *Helicobacter pylori*

**DOI:** 10.3329/jhpn.v29i5.8897

**Published:** 2011-10

**Authors:** Collise Njume, Anthony J. Afolayan, Amidou Samie, Roland N. Ndip

**Affiliations:** ^1^Microbial Pathogenicity and Molecular Epidemiology Research Group, Department of Biochemistry and Microbiology, University of Fort Hare, P/Bag X1314, Alice 5700, South Africa; ^2^Phytomedicine Research Group, Department of Botany, Faculty of Science and Agriculture, University of Fort Hare, P/Bag X1314, Alice 5700, South Africa; ^3^Department of Microbiology, University of Venda, Thohoyandou 0950, South Africa; ^4^Department of Biochemistry and Microbiology, Faculty of Science, University of Buea, Box 63, Buea, Cameroon

**Keywords:** Acetone, Antibiotic resistance, *Combretum molle*, Crude extracts, *Helicobacter pylori*, Microbial sensitivity tests, Minimum inhibitory concentration, South Africa

## Abstract

Infection with *Helicobacter pylori* is strongly associated with a number of gastroduodenal pathologies. Antimicrobial resistance to commonly-used drugs has generated a considerable interest in the search for novel therapeutic compounds from medicinal plants. As an ongoing effort of this search, the susceptibility of 32 clinical strains of *H. pylori* and a reference strain—NCTC 11638—was evaluated against five solvent extracts of *Combretum molle*, a plant widely used for the treatment of gastric ulcers and other stomach-related morbidities in South Africa. The extracts were screened for activity by the agar-well diffusion method, and the most active one of them was tested against the same strains by micro-broth dilution and time kill assays. Metronidazole and amoxicillin were included in these experiments as positive control antibiotics. The solvent extracts all demonstrated anti-*H*. *pylori* activity with zone diameters of inhibition between 0 and 38 mm. The most potent anti-*H*. *pylori* activity was demonstrated by the acetone extract, to which 87.5% of the clinical strains were susceptible. The minimum inhibitory concentration (MIC_90_) values for this extract ranged from 1.25 to 5.0 mg/mL while those for amoxicillin and metronidazole ranged from 0.001 to 0.94 mg/mL and from 0.004 to 5.0 mg/mL respectively. The acetone extract was highly bactericidal at a concentration of 2.5 and 5.0 mg/mL, with complete elimination of the test organisms in 24 hours. Its inhibitory activity was better than that of metronidazole (p<0.05) as opposed to amoxicillin (p<0.05). The results demonstrate that *C*. *molle* may contain therapeutically-useful compounds against *H*. *pylori*, which are mostly concentrated in the acetone extract.

## INTRODUCTION

*Helicobacter pylori* is a gram-negative microaerophilic spiral-shaped bacillus that affects the gastric mucosa and can be found attached to epithelial cells of the human stomach (1). Infection with this organism is strongly associated with chronic gastritis, peptic ulcer, duodenal ulcer, gastric adenocarcinoma, and mucosa-associated lymphoid tissue (MALT) lymphoma (2). The stomach of half of the world's population is colonized by this organism (3). Treatment of *H*. *pylori* infection is relatively successful, with up to 90% of patients exhibiting eradication of the organism with current therapeutic regimens (4). These regimens typically involve the use of a proton pump inhibitor (PPI) or bismuth compounds in combination with two antibiotics—most commonly amoxicillin and clarithromycin, or metronidazole (5). However, *H*. *pylori* infection continues to be difficult to eradicate with failure rates of up to 40% (6). A major factor to this failure is the development of antibiotic-resistant strains (5). It is, therefore, not uncommon to find other stronger antibiotics, particularly of the fluoroquinolone group, being part of the treatment regimen (7) but *H*. *pylori* is also developing resistance to these drugs (8). Considering that eradication therapies can be ineffective and undesirable side-effects may occur (9), the search for alternative therapeutic sources for the development of new anti-*H*. *pylori* compounds is imperative.

Other factors, including poor compliance by patients, cost of combination therapy, the location of the organism in the stomach, and the non-availability of drugs in some rural settings in Africa, are contraindications for some patients (3,10). Equally important is the increasing prevalence of virulent strains, particularly those expressing the cytotoxin-associated gene A antigen (*CagA*) associated with severe pathological conditions (11) that may be difficult to manage and post-therapeutic antibiotic resistance which has been known to decrease the cure rate by more than 50% (12). These factors have generated a considerable interest in the search for alternative treatment regimens against this notorious pathogen. Alternative therapeutic agents with highly-selective antibacterial activity against the organism, without the risk of resistance or other untoward effects, are necessary (5).

Medicinal plants are among the attractive sources of new drugs and have been used for treating gastrointestinal diseases and other ailments, particularly in the developing world where infectious diseases are endemic and modern health facilities are not always adequate or accessible (13). Antimicrobial compounds from plants may inhibit bacterial growth by mechanisms different from presently-used treatment regimens and could, therefore, be of clinical value in the treatment of resistant bacteria, including *H*. *pylori*. In fact, our previous studies have documented that some medicinal plant extracts have antibacterial activity against *H*. *pylori* (14).

The genus Combretum is mainly tropical and consists of numerous species, including *Combretum molle*, *C*. *woodii*, *C*. *erythophyllum*, *C*. *Apiculatum,* and *C*. *mossambicense* (15,16). They consist of trees, climbers, and shrubs (16). Almost every part of these plants—roots, leaves, seeds, and stem barks—is used in African traditional medicine for the treatment of parasitic, bacterial and fungal infections (15,16). The stem bark of *C*. *molle*, a small graceful deciduous tree (3-13 m high) is popularly used in South Africa for the treatment of stomach pains, dysentery, gastric ulcers, abdominal disorders, and other illnesses (15). Studies on its antibacterial properties have produced promising results (15). It is found in the traditional medicine market where it is commercialized for medicinal purposes. Despite its traditional uses in the treatment of gastric ulcers and other stomach-related morbidities, the activity of this plant has not been investigated against *H*. *pylori*, a major cause of gastric ulcer. This is surprising particularly as the prevalence of this organism is reported to vary between 50% and 80% in South Africa (17,18), and an alarming resistance of 95.5% has been reported in South Africa for metronidazole, one of the antibiotics used in the treatment regimen of *H*. *pylori* infections (4). This study was, therefore, carried out to evaluate the antimicrobial activity of *C*. *molle* on drug-resistant isolates of *H*. *pylori.* The aim was to identify the potential sources of cheap starting materials for the synthesis of new drugs that could be cheap and readily available to help circumvent the problem of increasing antimicrobial resistance.

## MATERIALS AND METHODS

### Bacterial strains

We used 32 resistant strains of *H. pylori* isolated from gastric biopsies of patients with recurrent peptic ulcer infection, despite treatment with metronidazole and amoxicillin, undergoing endoscopy at the Livingstone Hospital, Port Elizabeth. A standard control strain—NCTC 11638—was also included. Isolation and identification was done following our previously-reported scheme (4). Briefly, biopsies were homogenized under aseptic conditions in 0.2 g/L of cysteine and 20% of glycerol in Brain heart infusion (BHI) broth (Oxoid, England). A loopful of the homogenate was plated on freshly-prepared Columbia agar base (Oxoid, England) supplemented with 6% horse blood and Skirrow's supplement (Oxoid, England) containing trimethoprim (2.5 mg), vancomycin (5 mg), cefsulodin (2.5 mg), and amphotericin (2.5 mg). Inoculated plates were incubated at 37 °C for five days under microaerophilic conditions (5-6% O_2_, 10% CO_2_, and 80-85% N_2_) (Anaerocult Basingstoke, Hampshire, England). The isolates were identified based on colony morphology, positive oxidase, urease, catalase tests, and amplification of the *glmM* gene. Confirmed isolates were suspended in eppendorf tubes containing 1 mL of BHI broth and 20% glycerol and stored at −80 °C until future use. Gastric biopsies were only collected from patients who had given consent and had not been on antibiotics, PPI, or bismuth salts for at least a week.

### Preparation of plant material

The stem bark of *C*. *molle* R. Br. Ex G. Don (Combretaceae) was harvested in the vicinity of the University of Venda, Limpopo province, and transported in plastic bags to the School of Biological Sciences, University of Fort Hare, where they were identified and vouchers were deposited in the school's herbarium (CNUFH05). The plant material was washed, air-dried for two weeks, and ground to fine powder using a blender (ATO MSE mix, England).

### Preparation of plant extracts

Exactly 300 g of dried plant material was macerated separately in 600 mL of concentrated ethyl acetate, acetone, ethanol, and methanol in large glass bottles (SIMAX, Czech Republic). Aqueous extracts were also prepared by soaking the same amount of plant material in tap water. The bottles were labelled and put in an orbital shaker for 48 hours. The plant extracts were centrifuged at 1,006.2 g for five minutes at 4 °C and filtered using a fritted filter funnel of pore size 60 Å. The procedure was repeated twice, and the three extracts were combined and evaporated to dryness under vacuum in a rotary evaporator (BUCHI rota vapour, Flavil/Schweiz, Switzerland). The filtrate obtained from the aqueous extract was lyophilized (19). The dried crude extracts were collected in clean glass Petri-dishes and left open in a biosafety class II cabinet (Durban, South Africa) for complete evaporation of residual solvents. A 2-g sample of each extract was used for the preliminary bioassay, and where possible, another 2 g or more was put in universal bottles and kept in the extract bank. Stock solutions were prepared by dissolving the extracts in 10% dimethyl sulphoxide (DMSO) or 80% acetone (neither DMSO nor acetone was inhibitory to the *H*. *pylori* strains at the tested concentrations).

### Screening of crude extracts for anti-***H***. ***pylori*** activity

This was done by the agar-well diffusion method as previously reported (20). Briefly, *H*. *pylori* inocula prepared at McFarland's turbidity standard 2 were plated onto BHI agar supplemented with 5% horse blood and Skirrow's supplement (Oxoid, England). The inocula were evenly spread on the plate. The plate was allowed to dry for about 15 minutes. Wells (6 mm in diameter) were punched into the agar using a sterile stainless steel borer. The wells were filled with 65 μL of the extract at 100 mg/mL. Sixty-five μL of 0.05 μg/mL of clarithromycin and 10% DMSO were included in all the experiments as positive and negative controls respectively. The plates were incubated under microaerophilic conditions (Anaerocult, Oxoid, UK) at 37 °C for 72 hours after which the diameters of zones of inhibition were measured in mm. The experiment was repeated once, and the mean zones were recorded. A zone diameter of ≥14 mm was used as breakpoint susceptibility for clarithromycin and the extracts; this was also used for calculating their percentage susceptibilities (1). A plate inoculated with a reference strain (NCTC 11638) of *H*. *pylori* was included in all the experimental runs.

### Determination of minimum inhibitory concentration (MIC_90_)

The acetone extract which was the only extract to which more than 50% of the strains were susceptible was chosen for further determination of MIC by the micro-broth dilution method as earlier reported (21). The test was performed in 96-well plates. The test extract was prepared at a concentration of 5.0 mg/mL and filtered through a 2.0-μm filter (Acrodisc Pall, MI, USA). Two-fold dilutions of the extract were made in the test wells in BHI broth supplemented with 5% horse serum and Skirrow's supplement (Oxoid, England). The final extract concentration ranged from 0.001 to 5.0 mg/mL. Twenty μL of an 18-hour old broth culture of *H*. *pylori* (McFarland's turbidity standard 2) suspension was added to 100 μL of extract-containing culture medium. Control wells were prepared with culture medium plus bacterial suspension and broth only respectively. Metronidazole and amoxicillin were run, alongside the extract at concentration ranges of 0.005-5.0 mg/mL and 0.001-1.25 mg/mL respectively. An automatic ELISA micro-plate reader (Tokyo, Japan) adjusted to 620 nm was used for measuring the absorbance of the plates. The plates were incubated at 37 °C for 72 hours under microaerophilic conditions (Anaerocult Basingstoke, Hampshire, England), and the absorbance was read again at 620 nm. The initial and the post-incubation absorbencies were compared to detect an increase or a decrease in bacterial growth. The lowest concentration of the test extract resulting in inhibition of 90% of bacterial growth was recorded as the MIC. The strains were considered susceptible to the control antibiotics if their MIC_90_ values were <0.002 mg/mL for amoxicillin and <0.008 mg/mL for metronidazole (1).

### Determination of rate of killing

The rate and extent of killing of *H*. *pylori* by the acetone extract of *C*. *molle* was determined as described by Akinpelu *et al*. (22) with slight modifications. The turbidity of an 18-hour old broth culture of *H*. *pylori* was standardized to 10^8^ CFU/mL. One mL of this suspension was added to 9 mL of BHI broth supplemented with 5% horse serum and Skirrow's reagents containing the extract at 0.625 mg/mL (MIC/2), 1.25 mg/mL (MIC), 2.50 mg/mL (2 MIC), and 5.0 mg/mL (4 MIC) in McCartney bottles (Oxoid, England). A negative control bottle was prepared with bacterial suspension and broth only. A 0.1-mL sample was plated from these bottles before incubation at 37 °C under microaerophilic conditions. Exactly 0.5 mL of each suspension was withdrawn at a six-hour interval for 72 hours and transferred to 4.5 mL of BHI broth recovery medium containing 3% ‘Tween 80’ to neutralize the effects of the antimicrobial extract carry-overs from the test organisms. The suspension was 10-fold serially diluted in sterile saline (0.9% w/v sodium chloride) and plated in triplicates. The plates were incubated at 37 °C for 72 hours under microaerophilic conditions, and the viable counts were determined.

### Statistical analysis

Results were expressed as mean±standard deviation using the SPSS software (version 17.0) (Chicago, Illinois, 2009) and Excel. One-way analysis of variance (ANOVA), followed by Turkey's *post-hoc* test, was used for comparing the mean difference in inhibitory activities of extracts and antibiotics. The differences were considered significant at p<0.05.

### Ethical approval

The Eastern Cape Department of Health and the Govan Mbeki Research and Development Centre, University of Fort Hare, approved the study.

## RESULTS

### Extract yield

The total amount of crude extract obtained with the different solvents showed that methanol was quantitatively the best solvent for extraction, with a crude extract yield of 5.1 g (1.7%), followed by acetone 4.6 g (1.5%), ethanol 3.2 g (1.1%), water 3.1 g (1.0%), and ethyl acetate 1.3 (0.4%). Ethyl acetate, acetone and methanol extracts were dark brown in colour while ethanol and aqueous extracts appeared as brown to light brown crystals.

### Antimicrobial susceptibility testing and determination of minimum inhibitory concentration

**Fig. 1.**
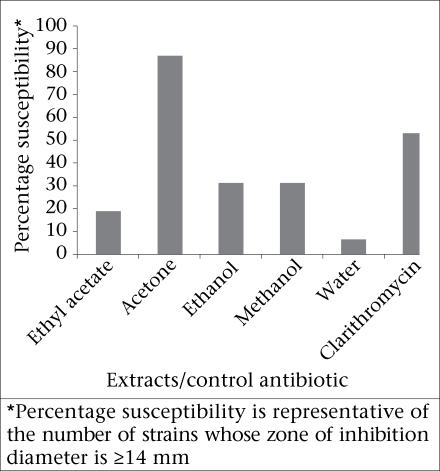
Anti-H. *pylori* activity of crude extracts of *C. molle* by agar well diffusion method

All the crude extracts tested in this study demonstrated antimicrobial activity with zone diameters of inhibition ranging from 0 to 38 mm (Table 1). The highest zone diameter of 38 mm was recorded for the acetone extract, to which 87.5% of the clinical strains were susceptible (Fig. 1). Zone diameters of inhibition for the acetone extract were significantly different from the other extracts (p<0.05) as opposed to the control antibiotic (p>0.05) (Table 1). Eleven (34.4%) and six (18.8%) of the 32 strains tested against the acetone and ethanol extracts respectively recorded susceptible zones of inhibition as opposed to the positive control which was resistant but the differences were not significant (p>0.05).

**Table 1.** Screening of crude extracts of *C. molle* against *H. pylori* isolates

Extract/ antibiotic	Mean zone diameter (mm)	Inhibition zone diameter range (mm)	p value					
			EA	A	E	M	H _2_ O	CLR
EA	10.7±4.7	0-21	-	0.00	0.62	0.054	0.00	0.28
A	17.5±5.0	10-38	0.00	-	0.02	0.03	0.00	0.10
E	13.0±4.7	7-35	0.62	0.02	-	1.00	0.00	0.99
M	13.1±5.3	7-32	0.54	0.03	1.00	-	0.00	0.99
H _2_ O	2.8±5.5	0-20	0.00	0.00	0.00	0.00	-	0.00
CLR	13.7±9.1	0-32	0.28	0.10	0.99	0.99	0.00	-

Data are mean±SD values of 33 independent determinations for each extract or control antibiotic. The mean difference was considered significant at p<0.05. A=Acetone;

CLR=Clarithromycin;

E=Ethanol;

EA=Ethyl acetate;

H _2_ O=Aqueous;

M=Methanol;

SD=Standard deviation;

-=No comparison

The MIC values for the acetone extract ranged from 1.25 to 5.0 mg/mL while those for amoxicillin and metronidazole ranged from 0.001 to 0.94 mg/mL and 0.004 to 5.0 mg/mL respectively (Table 2).

### Bactericidal activity

**Fig. 2.**
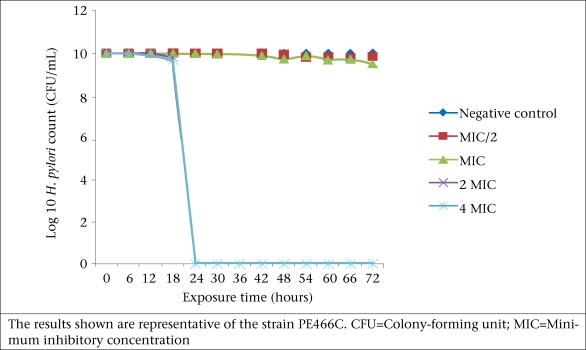
Effect of crude acetone extracts of *C. molle* on the growth of *H. pylori*

The acetone extract of *C*. *molle* exhibited considerable bactericidal activity against the isolates at all concentrations tested over a 72-hour period. The test organisms were completely eliminated at a concentration of 2.5 and 5.0 mg/mL within 24 hours (Fig. 2). No cells were killed in the first six hours of the experiment at all the extract concentrations tested.

## DISCUSSION

Post-therapeutic antibiotic-resistant *H*. *pylori* reduces the cure rate of infection by up to 66% (12). With very few exceptions, the most commonly-recommended treatment regimen (PPI, amoxicillin, and clarithromycin or metronidazole) now provides unacceptably low treatment successes (23), with approximately one in five patients requiring second and third-line therapies due to eradication failure (12). It should, therefore, not be surprising that all the clinical strains tested in this study were resistant to metronidazole and amoxicillin considering that they were isolated from patients who had been on treatment regimens involving these drugs (4). We were, therefore, probably dealing with a small group of patients with a high level of post-therapeutic resistance confirmed by the high MIC values observed.

Other studies have reported high metronidazole and amoxicillin resistance in the developing world (1,4,5,24). Metronidazole resistance is an existing problem in this part of the world and is probably due to drug pressure because this inexpensive drug is also used in the treatment of gynaecological problems and protozoa infections (1,4) while amoxicillin resistance can be attributed to the non-adherence to drug-prescription protocols. Consequently, an increased eradication failure will be observed with treatment regimens involving these drugs. Antimicrobial susceptibility testing to establish resistance patterns, therefore, becomes imperative to guide empiric treatment. Other measures, such as strict antibiotic restriction practices, education of the population, and pharmacovigilance will go a long way to improve the management of antibiotic use in the developing world.

Our results showed that methanol was quantitatively the best solvent for extraction. Different solvents may be employed in the extraction of antimicrobial compounds from plants; however, the success in the isolation process is largely dependent on the type of solvent used (25). The good extracting ability of methanol recorded in this study corroborates findings of other studies (25-27), thus confirming this solvent as a good extractant of phytochemicals from *Combretum* species and other plants. However, the quantity of extract may not always relate proportionately to the activity as revealed by the low anti-*H*. *pylori* activity of the methanol extract recorded herein. Nevertheless, extracts with little or no activity *in vitro* may have properties similar to pro-drugs which are administered in an inactive form. Their metabolites could be active *in vivo* (14).

**Table 2.** Minimum inhibitory concentration (90%) of *C. molle* and control antibiotics (mg/mL)

*H. pylori* strain	MIC _90_ (mg/mL)		
	Acetone extract	Metronidazole	Amoxicillin
PE2A	1.25	2.50	0.02
PE5A	2.5	–	–
PE9C	5	–	0.02
PE11A	1.25	–	0.94
PE11C	1.9	–	0.94
PE14C	1.25	–	0.02
PE26A	2.5	–	0.02
PE70A	2.5	–	0.02
PE76A	1.9	2.5	0.02
PE84C	1.9	–	0.02
PE93A	1.25	5	0.02
PE93C	2.5	–	0.02
PE102C	2.5	–	0.02
PE115A	2.5	5	0.02
PE155A	–	1.9	0.02
PE162A	2.5	5	0.1
PE219A	1.25	1.9	0.02
PE252C	1.25	1.9	0.02
PE258C	–	2.5	0.02
PE265C	2.5	–	–
PE296C	1.25	5	0.02
PE308C	5	5	0.02
PE369A	2.5	–	0.02
PE369C	2.5	–	0.63
PE397C	1.25	5	0.02
PE402A	2.5	5	0.02
PE411A	1.9	–	0.63
PE411C	1.9	–	0.02
PE430A	2.5	–	0.02
PE430C	2.5	–	0.02
PE435A	2.5	–	0.94
PE466C	1.25	1.9	0.02
NCTC 11638	2.5	0.004	0.001
Mean±SD	2.2±0.9	3.3±1.7	0.2±0.3

–=Value not within susceptible range. The results shown are representative of 33 independent experiments for each extract or control antibiotic and duplicate determinations for each strain.

MIC=Minimum inhibitory concentration;

SD=Standard deviation

The results of this study indicate that the ethanol and methanol extracts were only minimally active (Fig. 1). However, in a study on rats in Brazil by Nunes *et al*. (28), the ethanolic extract of the stem bark of a local *Combretum* species—*C*. *leprosum*—exhibited anti-ulcerogenic and gastro-protective effects by increasing the volume and pH of gastric juice while decreasing the acid output (28). Although Nunes *et al.* did not investigate the antimicrobial activity of their extracts against *H*. *pylori* (28), the effects demonstrated by the crude ethanolic extract of *C*. *leprosum* in the animal model could be useful in preventing the development of severe pathological conditions in an *H*. *pylori*-infected mucosa.

The zone of inhibition diameters and percentage susceptibilities seem to decrease with increase in polarity of the solvent from ethanol to water (Fig. 1), which may imply that the isolates were not very sensitive to the polar compounds of this plant or at least, not many anti-*H. pylori* compounds were extracted by polar solvents. However, the activity demonstrated by these extracts indicates their potential as useful bioactive substances.

The acetone extract demonstrated remarkable activity against the test strains in the entire study. This indicates that the active components of this plant are more soluble in acetone and have a good antimicrobial potential. Further investigation may lead to the isolation of potentially-useful compounds for the treatment of *H*. *pylori* infections. The activity of this extract compared favourably with metronidazole, with no significant differences between their mean MIC values (p>0.05). This is remarkable considering that all the clinical strains used in this study were resistant to the antibiotics.

The extract was also strongly bactericidal at a concentration of 2.5 and 5.0 mg/mL, with complete elimination of the organisms in 24 hours (Fig. 2). However, active components in the crude extract may be acting in synergism to produce greater antimicrobial effects (29). In any case, the acetone extract of *C*. *molle* may be considered a possible new source of compound**s** for the management of infections caused by resistant strains of *H*. *pylori*. This is particularly important in the study area given the current trend and ever-evolving nature of resistant *H*. *pylori* (4).

Acetone is used for extracting mostly flavonoids and steroids (15,30,31). Flavonoids are known to be synthesized by plants in response to microbial infection (32,33), which may account for their antimicrobial activity against a wide range of organisms.

Several studies have reported on the good antimicrobial activity of the ethyl acetate fraction of *Combretum* species (34,35) but *H*. *pylori* was not among the organisms tested. Our findings provide preliminary evidence that the ethyl acetate extract of *C*. *molle* has very little activity against *H*. *pylori*. The aqueous extracts of *C*. *molle* also showed very little activity (6.3%) against *H*. *pylori* (Fig. 1). Other studies have also reported very low antimicrobial activity of the aqueous extract of other members of the Combretaceae and other plants (15,35). Despite its availability and relatively low toxicity, water may still not be a suitable solvent for the extraction of anti-*H*. *pylori* compounds from *C*. *molle*.

### Conclusions

The results of the study provide preliminary scientific validation of the traditional medicinal use of this plant in the treatment of infections symptomatic of *H*. *pylori*. This plant may contain compounds, mostly in the acetone crude extract that could be used as lead molecules for the synthesis of novel drugs against recurrent and resistant *H*. *pylori* infections. Isolation and characterization of the biologically-active constituents of the acetone extract of *C*. *molle* and a detailed assessment of their *in-vivo* potencies will add more value to their potential usefulness as anti-*H*. *pylori* agents. These aspects are already receiving attention in our group.

## ACKNOWLEDGEMENTS

The authors are grateful to the National Research Foundation, South Africa (Grant No. CSUR 2008052900010) and the Govan Mbeki Research and Development Centre, University of Fort Hare, South Africa, for funding the study. They thank Dr. Naidoo N, Dr. Tanih NF, Mr. Okeleye BI, and Mr. Tshikhawe P for technical assistance.

## References

[1] Ndip RN, Malange TAE, Ojongokpoko JEA, Luma HN, Malongue A, Akoachere JFK (2008). *Helicobacter pylori* isolates recovered from gastric biopsies of patients with gastro-duodenal pathologies in Cameroon: current status of antibiogram. Trop Med Inter Health.

[2] Adeniyi CB, Lawal TO, Mahady GB (2009). *In vitro* susceptibility of *Helicobacter pylori* to extracts of *Eucalyptus camaldulensis**Eucalyptus torelliana*. Pharmaceut Biol.

[3] Romano M, Cuomo A (2004). Eradication of *Helicobacter pylori*: a clinical update. Med Gen Med.

[4] Tanih NF, Okeleye BI, Naidoo N, Clarke AM, Mkwetshana N, Green E (2010). Marked susceptibility of South African *Helicobacter pylori* strains to ciprofloxacin and amoxicillin: clinical implication. S Afr Med J.

[5] Njume C, Afolayan AJ, Ndip RN (2009). An overview of antimicrobial resistance and the future of medicinal plants in the treatment of *Helicobacter pylori* infections. Afr J Pharm Pharmacol.

[6] Lai CH, Kuo CH, Chen PY, Poon SK, Chang CS, Wang WC (2006). Association of antibiotic resistance and higher internalization activity in resistant *Helicobacter pylori* isolates. J Antimicrob Chemother.

[7] Malfertheiner P, Megraud F, O'Morain C, Bazzoli F, El-Omar E, Graham D (2007). Current concepts in the management of *Helicobacter pylori* infection: the Maastricht III Consensus Report. Gut.

[8] Eisig JN, Silva FM, Barbuti RC, Rodriguez TN, Malfertheiner P, Moraes Filho JP (2009). Efficacy of a 7-day course of furazolidone, levofloxacin, and lansoprazole after failed *Helicobacter pylori* eradication. BMC Gastroenterol.

[9] Aboderin OA, Abdu AR, Odetoyin B, Okeke IN, Lawal OO, Ndububa DA (2007). Antibiotic resistance of *Helicobacter pylori* from patients in Ile-Ife, South-West, Nigeria. Afr Health Sci.

[10] Me´Graud F, Lehours P (2007). *Helicobacter pylori* detection and antimicrobial susceptibility testing. Clin Microbiol Rev.

[11] Tanih NF, McMillan M, Naidoo N, Ndip LM, Weaver LT, Ndip RN (2010). Prevalence of *Helicobacter pylori* VacA, CagA and iceA genotypes in South African patients with upper gastrointestinal diseases. Acta tropica.

[12] Bohr UR, Malfertheiner P (2009). Eradication of *H. pylori* infection: the challenge is on if standard therapy fails. Therapeut Advan Gastroenterol.

[13] Samie A, Obi CL, Bessong PO, Namrita L (2005). Activity profiles of fourteen selected medicinal plants from rural Venda communities in South Africa against fifteen clinical bacterial species. Afr J Biotechnol.

[14] Ndip RN, Malange Tarkang AE, Mbullah SM, Luma HN, Malongue A, Ndip LM (2007). In vitro anti-*Helicobacter pylori* activity of extracts of selected medicinal plants from North West Cameroon. J Ethnophamacol.

[15] Eloff JN, Katerere DR, Mcgaw LJ (2008). The biological activi-ty and chemistry of the southern African Combretaceae. J Ethnopharmacol.

[16] Elegami AA, Osman SM, Omer ME, Ishag KM (2007). In-vitro-antibacterial activity of some Sudanese *Combretum* species. Inter J Trop Med.

[17] Samie A, Obi CL, Barrett LJ, Powell SM, Guerrant RL (2007). Prevalence of *Campylobacter* species, *Helicobacter pylori**Arcobacter* species in stool samples from the Venda region, Limpopo, South Africa: studies using molecular diagnostic methods. J Infect Dis.

[18] Dube C, Nkosi TC, Clarke AM, Mkwetshana N, Green E, Ndip RN (2009). *Helicobacter pylori* antigenemia in an asymptomatic population of the Eastern Cape province, South Africa: public health implications. Rev Environ Health.

[19] Castillo-Juárez I, González V, Aime-Aguilar H, Martínez G, Linares E, Bye R (2009). Anti-*Helicobacter pylori* activity of plants used in Mexican traditional medicine for gastrointestinal disorders. J Ethnopharmacol.

[20] Boyanova L, Gergova G, Nikolov R, Derejian S, Lazarova E, Katsarov N (2005). Activity of Bulgarian propolis against 94 *Helicobacter pylori* strains in vitro by agar-well diffusion, agar dilution and disc diffusion methods. J Med Microbiol.

[21] Bonacorsi C, Raddi MS, Carlos IZ, Sannomiya M, Vilegas W (2009). Anti-*Helicobacter pylori* activity and immunostimulatory effect of extracts from *Byrsonima crassa* Nied. BMC Complement Alternat Med.

[22] Akinpelu DA, Aiyegoro AO, Okoh AI (2009). Studies on the biocidal and cell membrane disruption potentials of stem bark extracts of *Afzelia africana* (Smith). Biol Res.

[23] Graham D, Fischbach L (2010). *Helicobacter pylori* treatment in the era of increasing antibiotic resistance. Gut.

[24] Smith SI, Oyedeji KS, Arigbabu AO, Atimomo C, Coker AO (2001). High amoxicillin resistance in *Helicobacter pylori* isolated from gastritis and peptic ulcer patients in Western Nigeria. J Gastroenterol.

[25] Masoko P, Picard J, Eloff JN (2007). The antifungal activity of twenty-four Southern African *Combretum* species (Combretaceae). S Afr J Bot.

[26] Fyhrquist P, Mwasumbi L, Hæggström CA, Vuorela H, Hiltunen R, Vuorela P (2002). Ethnobotanical and antimicrobial investigation on some species of *Terminalia**Combretum* (Combretaceae) growing in Tanzania. J Ethnopharmacol.

[27] Ezekiel CN, Anokwuru CP, Nsofor E, Odusanya OA, Adebanjo O (2009). Antimicrobial activity of the methanolic and crude alkaloid extracts of *Acalypha wilkesiana* cv. macafeeana copper leaf. Res J Microbiol.

[28] Nunes PH, Cavalcanti PM, Galvão SM, Martins MC (2009). Antiulcerogenic activity of. Combretum leprosum. Pharmazie.

[29] Eloff JN (1998). Which extractant should be used for the screening and isolation of antimicrobial components from plants?. J Ethnopharmacol.

[30] Cowan MM (1999). Plant products as antimicrobial agents. Clin Microbiol Rev.

[31] Afolayan AJ, Lewu FB (2009). Antimicrobial activity of. Alepidea amatymbica. Pharmaceut Biol.

[32] Hernández NE, Tereschuk ML, Abdala LR (2000). Antimicrobial activity of flavonoids in medicinal plants from Tafí del Valle (Tucumán, Argentina). J Ethnopharmacol.

[33] Schinor EC, Salvador MJ, Ito IY, Dias DA (2007). Evaluation of the antimicrobial activity of crude extracts and isolated constituents from. Chresta scapigera. Brazil J Microbiol.

[34] Onocha PA, Audu EO, Ekundayo O, Dosumu OO (2005). Phytochemical and antimicrobial properties of extracts of. Combretum racemosum. Acta Hort.

[35] Eloff JN, Famakin JO, Katerere DRP (2005). *Combretum woodii* (Combretaceae) leaf extracts have high activity against gram-negative and gram-positive bacteria. Afr J Biotechnol.

